# A signal-detection account of item-based and ensemble-based visual change detection: A reply to Harrison, McMaster, and Bays

**DOI:** 10.1167/jov.24.2.10

**Published:** 2024-02-26

**Authors:** Daniil Azarov, Daniil Grigorev, Igor Utochkin

**Affiliations:** 1 HSE University, Moscow, Russia; 2 Koç University, Istanbul, Turkey; 3 University of Chicago, Chicago, IL, USA

**Keywords:** visual working memory, change detection, ensemble encoding, signal detection theory, optimal summation

## Abstract

Growing empirical evidence shows that ensemble information (e.g., the average feature or feature variance of a set of objects) affects visual working memory for individual items. Recently, Harrison, McMaster, and Bays (2021) used a change detection task to test whether observers explicitly rely on ensemble representations to improve their memory for individual objects. They found that sensitivity to simultaneous changes in all memorized items (which also globally changed set summary statistics) rarely exceeded a level predicted by the so-called optimal summation model within the signal-detection framework. This model implies simple integration of evidence for change from all individual items and no additional evidence coming from ensemble. Here, we argue that performance at the level of optimal summation does not rule out the use of ensemble information. First, in two experiments, we show that, even if evidence from only one item is available at test, the statistics of the whole memory set affect performance. Second, we argue that optimal summation itself can be conceptually interpreted as one of the strategies of holistic, ensemble-based decision. We also redefine the reference level for the item-based strategy as the so-called “minimum rule,” which predicts performance far below the optimum. We found that that both our and Harrison et al. (2021)’s observers consistently outperformed this level. We conclude that observers can rely on ensemble information when performing visual change detection. Overall, our work clarifies and refines the use of signal-detection analysis in measuring and modeling working memory.

## Introduction

Working memory is usually described as a limited-capacity system that stores the small amount of information necessary to perform the current task (Baddeley, 1986; Cowan, 2001; Miller, 1956). Existing theories and related methodology often characterize this visual part of this system and its limitations in terms of the number of items that can be stored (Cowan, 2001; Luck & Vogel, 1997; Alvarez & Cavanagh, 2004; Awh, Burton & Vogel, 2007; Cowan, Chen, & Rouder, 2004) and the quality with which these items can be stored (Wilken & Ma, 2004; Zhang & Luck, 2008, Zhang & Luck, 2009). Although the object is often considered a natural representational unit of visual working memory, there is a debate about whether visual working memory stores discrete objects in a limited number of slots (Luck & Vogel, 1997; Zhang & Luck, 2008) or flexibly allocates processing resources between various numbers of item representations at cost of quality as a function of stimuli and task (Ma, Husain, & Bays, 2014; but see Ngiam, 2023, for notes on a broader theory map encompassing both views).

However, not only objects and their individual properties appear to define visual working memory. Relations between different objects in physical and feature spaces can also play a role in structuring working memory representations (Brady & Alvarez, 2015b; Brady & Tenenbaum, 2013; Jiang, Olson, & Chun, 2000; Orhan & Jacobs, 2013). A series of studies of the past decade suggests that ensemble representations that convey coarse statistical information about all items together, such as the average feature or feature variability, can also contribute to visual working memory measured for individual objects. Brady and Alvarez (2011) showed that individual sizes of remembered items tended to be biased toward the overall mean size of the display and toward the local mean of a color group the item belonged to. Similarly, Corbett (2017) found that objects from the same Gestalt group were reported with correlated errors, in contrast to objects from different groups. Brady and Alvarez (2015a) found that color reports from visual working memory highly varied across individual displays (although they were quite consistent across observers). Importantly, Brady and Alvarez also showed that report errors observers made for particular colors they correlated with ensemble properties of those displays, namely, how variable colors were overall and which colors were similar or dissimilar to each other. Utochkin and Brady (2020) showed that, as feature variability of memoranda increased, both ensemble and individual items were reproduced with a greater error suggesting that variance information is used to report individuals.

In their recent study, Harrison, McMaster, and Bays (2021) sought to test whether the previously reported ensemble effects on individual item reports could be explained without assuming any ensemble representations being held in visual working memory. In a series of experiments, their observers performed a change detection task. In each trial, the observers were briefly shown two or four discs of different colors or Gabor patches with different orientations which the observers had to remember. After a blank interval, the observers were shown either one randomly chosen item (one-item condition) or all items (full-set condition) that either changed or stayed the same as at the beginning. Observers had to answer whether they had seen a change. If the change was present in the full-set condition, then all items changed either in the same or opposite directions in a feature space, making the post-change set shift in mean, variance, or both. To measure performance, Harrison et al. (2021) used the standard sensitivity index from signal detection theory, *d′*:

(1) $$\begin{array}{c} d^{'}=z\left(H\right)-z\left(\mathrm{FA}\right),\; \end{array}$$

where *z*(H) and *z*(FA) are *z*-scores of a hit probability (answering “yes” given that the change is present) and a false alarm probability (answering “yes” given that the change is absent). Here the *d′* can be interpreted as a measure of separation between the distributions of *evidence for change* when change is present (signal distribution) and when change is absent (noise distribution).

Because only one post-change item remained on the screen in the one-item condition, Harrison et al. (2021) assumed that performance in this condition can be used as a baseline measure of change detection based only on individual item memories. With that assumption in mind, performance in the full-set condition can be predicted for an ideal observer who independently samples evidence for each of the items and uses the sum of these samples without calculating any ensemble summaries. This prediction comes from the multidimensional version of signal detection theory (SDT). Because each item change is assumed to initiate an independent (orthogonal) sampling process constrained by the its own signal-noise separability (*d′*_i_*)*, then the full-set discriminability (*d′*_total_*)* can be expressed using the Pythagorean theorem, as follows (Hautus, Macmillan, & Creelman, 2022):

(21) $$\begin{array}{c} d{'}_{total}=\sqrt{\sum_{i=1}^{n}d_{i}^{'2}},\; \end{array}$$

where *n* is the number of items in the full set. This equation implies that *d*′_total_ is a distance between two *n*-variate distributions, one corresponding to all items being sampled from *n* noise distributions (no-change condition) and another corresponding to all items being sampled from their respective signal distributions (change in all *n* components). As Harrison et al. (2021) suggest, Equation 2a predicts the *d′*_total_ if change detection is based on *optimal summation* of evidence only from individual items. On the other hand, *d′*_total_ based on the summation of evidence from individual items can be defined via the distribution of sums of independent samples drawn from each of the *n* distributions (we will refer to it as to the statistical solution). Defined this way, *d′*_total_ is the sum of individual *d′* (*d′*_i_*)* normalized by their pooled standard deviation (assuming the standard deviation of each individual item's distribution is 1):

(22) $$\begin{array}{c} d{'}_{total}=\frac{\sum_{i=1}^{n}d{'}_{i}}{\sqrt{n}}.\; \end{array}$$

It is easy to see that, if change magnitude is the same for each item (which was the case in those trials of Harrison et al. (2021) experiments where mean color or orientation changed), then the predicted *d′*_total_ can be found using a single one-item *d′* (*d′*_one_) measured for any of the items:

(3) $$\begin{array}{c} d{'}_{total}=\sqrt{n}\cdot d{'}_{one}.\; \end{array}$$

If ensemble information is additionally used to evaluate evidence for change then observers should outperform the optimal summation model in the full-set condition. This prediction also follows from the multidimensional SDT. If the observer optimally sums evidence from *n* individual items and from ensemble statistics (e.g., tracks if the average color changes across the displays) then ensemble statistics should form at least one more axis in the evidence space. This additional axis should yield an additional benefit to the *d′*_total_, provided non-zero ensemble sensitivity (*d′*_ensemble_). Harrison et al. (2021) express this in the following Pythagorean theorem equation:

(41) $$\begin{array}{c} d{'}_{total}=\sqrt{n\cdot {\left(d{'}_{one}\right)}^{2}+{\left(d{'}_{ensemble}\right)}^{2}}.\; \end{array}$$

The statistical reinterpretation of this equation can be written as follows:

(42) $$\begin{array}{c} d{'}_{total}=\frac{n\cdot d{'}_{one}+d{'}_{ensemble}\cdot \sigma_{ensemble}}{\sqrt{n+\sigma_{ensemble}^{2}}},\; \end{array}$$

where σ_ensemble_ is the standard deviation of the ensemble noise distribution in proportion to the individual item's noise used as the unit of the discriminability space.

Harrison et al. (2021) did not find evidence for such benefit in most of their data, although there were some minor exceptions. Specifically, the authors reported observers outperformed optimal summation for full sets changing in variance when discrimination was difficult and feature variance was low (that is, when items were similar). Based on these findings, Harrison et al. (2021) concluded that the role of ensemble statistics in visual change is limited.

Because the conclusion about limited memory for ensemble information in Harrison et al. (2021) is based on testing their data against the optimal summation model, we decided to have a closer look at this model in terms of its capability to dissociate “no-ensemble” change-detection from that relying on some ensemble information and to evaluate the contribution of ensemble memory. Specifically, we point out three caveats challenging the interpretation of this model as a “no-ensemble” model. These caveats concern some of the model assumptions and conceptual interpretations.

### Is the single-item *d′* always a measure of ensemble-free memory?

One critical assumption the model by Harrison et al. (2021) rests upon is that the *d′* measured in one-item change detection (*d′*_one_) represents ensemble-free working memory performance. Indeed, if the observer memorizes a set of items and then sees a single test item, the observer cannot estimate a change in an ensemble statistic of the whole set. However, that does not automatically imply that the observer cannot compare the test item to the ensemble representation of the memory set, even if and especially if it is not obvious whether this individual item has changed: “I do not remember whether this particular disc has changed its color but now it looks redder than all original items on average.” If this is the case, then the observed *d′*_one_ combines a component coming from that item and another component coming from an ensemble. Therefore the *d′*_one_ is not necessarily a baseline for perfectly independent representations of individual items. In the present study, we will show that variation in ensemble properties of a memory set and the direction of change relative to the original feature distribution affect the *d′*_one_ in both one-item and full-set change detection.

It is also important to note that ensemble representations are usually defined as representations of relatively similar objects (Alvarez, 2011; Corbett, Utochkin, & Hochstein, 2023; Whitney & Yamanashi Leib, 2018). The strength of the ensemble effect should depend on similarity. If all features fit within a reasonable range then they make an ensemble that can be well described by the same set of summary statistics. By contrast, unitary ensemble statistics can be useless for dissimilar sets. For example, if you look at a bush with a lot of greenish, large and oval leaves and a lot of small, reddish, and round berries, there is no use to represent two obviously categorical subsets as one ensemble. Indeed, there is evidence from the ensemble and texture perception literature that sets of highly dissimilar items fall apart into locally similar clusters (Treue, Hol, & Rauber, 2000; Utochkin, 2015; Im, Tiurina, & Utochkin, 2021). This is also true for clustering in working memory (Lew & Vul, 2015; Son, Oh, Kang, & Chong, 2020). Utochkin and Brady (2020) systematically manipulated similarity and found that ensemble domination is stronger with more similar items, as the relative bias to the mean was stronger if the items had more similar orientations. Brady and Alvarez (2015) generated random colors for sample stimuli but their model carefully took into account how individual colors clustered by similarity. When Harrison et al. (2021) broke down their data into those coming from low-variance and high-variance displays, they also found some evidence for potential ensemble influence provided by the low-variance displays (yet this evidence was found in two out of six experiments and cannot be considered robust). The role of feature similarity in driving the potential ensemble effects will be critical for our experimental manipulations in the current work.

### What limits the effective contribution of ensemble summary statistics

As follows from Equations 4a and 4b, the ensemble-based component *d′*_ensemble_ of change detection can effectively elevate performance above the level optimal summation (Equation 3) if the ensemble dimension is orthogonal to individual-item dimensions in the multidimensional evidence space. This implies that ensemble information should be sampled independently from information used to represent individual items. In other words, the observer has to sample each pre-change and post-change item twice: One set of samples is to encode individual features and another is to encode ensemble summaries. Although such double sampling is not prohibited by the formal model, it appears not a very parsimonious and plausible strategy for working memory, given its limitations. On the other hand, if the item-based and ensemble-based sources of evidence come from the same set of samples, then the sum of individual changes and the ensemble change are perfectly correlated, such that predicted *d′*_total_ would always be at the level of optimal summation and can never exceed it. In other words, the model presented in Equation 4 in fact cannot distinguish between optimal summation with and without ensemble summary.

**Figure 1.**
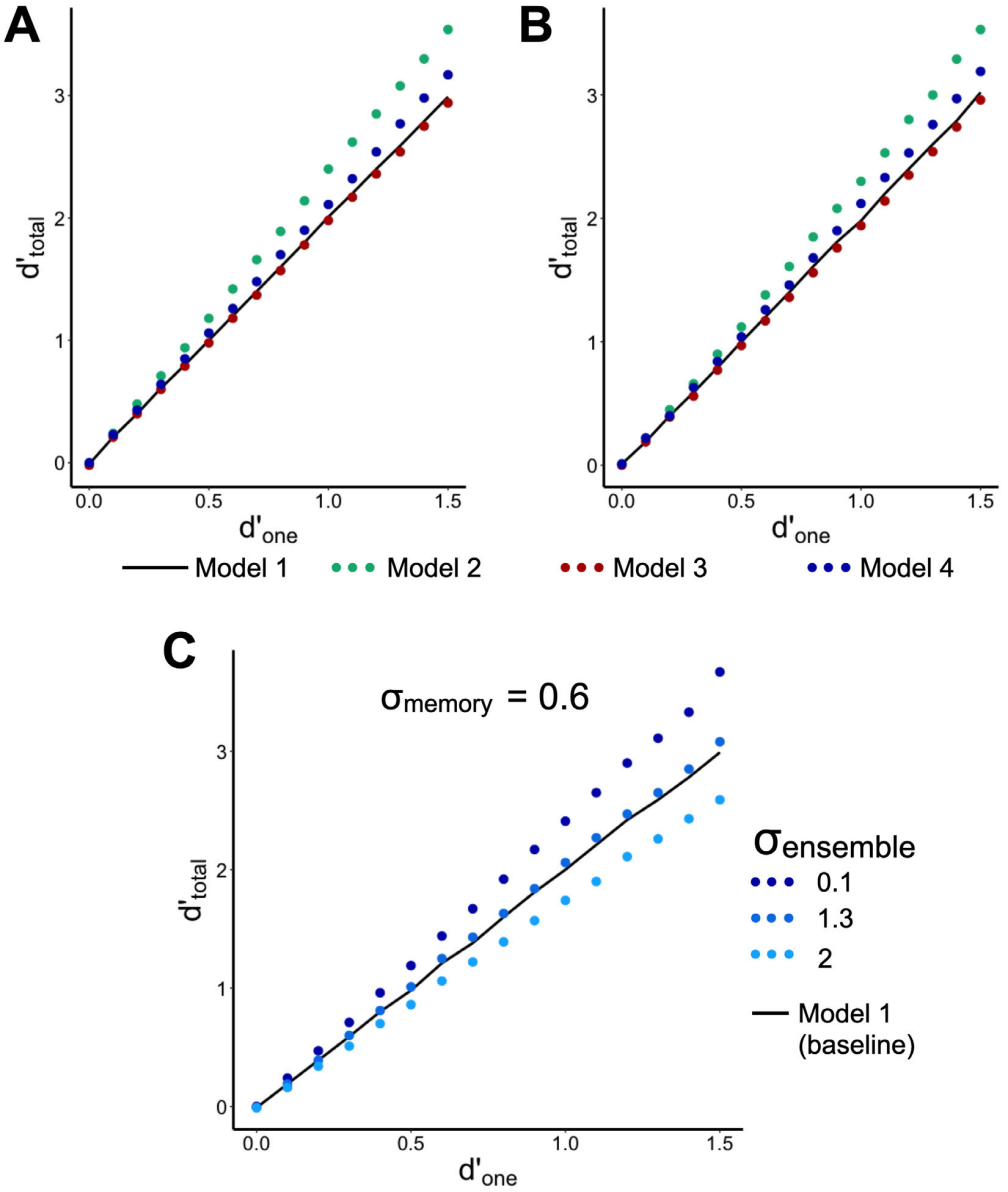
Expected *d′*_total_ as a function of *d′*_one_ in full-set change detection with set size 4, as in Harrison et al. (2021), when (**A**) mean or (**B**) variance of the post-change set changes. Predictions for four models are presented in (**A**) and (**B**): Model 1: only optimal summation with no ensemble memory (OS), Model 2: optimal summation with ensemble memory and double sampling; Model 3: optimal summation with ensemble memory and single sampling, and Model 4: optimal summation with ensemble memory, single sampling and independent memory noise (σ_memory_ = 0.6 *z*-units of the evidence-for-change distributions) in addition to sampling noise. Panel (**C**) illustrates Model 5: optimal summation with ensemble memory, single sampling, and two sources of noise independent from sampling, memory noise applied to individual items (σ_memory_) and integration noise applied to ensemble summaries (σ_ens_), only in mean-change trials. Different dotted lines in (**C**) illustrate that the use of ensemble statistics can cause benefit, no effect, or loss compared to the level of optimal summation (Model 1), depending on the ratio between the different sources of noise. That highlights a challenge for Harrison et al. (2021) approach to generate diagnostic behavioral criterion of using versus not using ensemble summary statistics.

Figure 1 illustrates how the expected benefit from ensemble information can differ from optimal summation as a function of assumption regarding sampling. This figure is a result of a computational simulation of change detection in four-item displays with stimulus manipulations similar to those implemented by Harrison et al. (2021). Specifically, we directly simulated sampling individual features from pre-change and post-change displays, computation of ensemble summary statistics, evidence integration with or without ensemble, and decision making based on the integrated evidence (for more details of the simulation, see Appendix). We varied the *d′*_one_ as a model input and obtained the *d′*_total_ as a model output. As Figures 1A and 1B show, the model with ensemble memory and double sampling indeed predicted the boost in performance relative to the model with no ensemble memory, whereas the model with ensemble memory and single sampling performed at the same level as the model with no ensemble memory.

Having said that, a more realistic psychophysical model should take into account sources of noise other than that coming from sampling individual items. For example, even if the pre-change items are sampled just once for representing both individuals and summary statistics, these representations can be further corrupted by independent noise added to each sample during memory delay (we will refer to it as to memory, σ_memory_). This additional independent noise should reduce the correlation between the optimally integrated evidence and evidence from ensemble summary statistics, which, in turn, might yield some benefit from using the summary statistics. On the other hand, some noise can be also applied at the integration stage (for example, when ensemble summary statistics are calculated). This integration noise (we can also add there a memory noise applied to the summary representation during retention and term everything “ensemble noise,” σ_ensemble_) counteracts the gain from applying the independent memory noise of the individual representations. The total amount of potential benefit from using ensemble summary statistics in addition to optimal summation, therefore, depends on (1) the ratio between the sampling noise and other sources of memory noise unrelated to sampling but applied to each individual item and (2) the ratio between the individual memory noise and ensemble noise that occurs when ensemble summaries are computed and stored in memory (Figure 1C).

These models can be formally represented as various individual cases of the following equation (which is Equation 4b with an additional term, covariation between the sum of individual evidence and ensemble summary statistics):

(5) $$\begin{array}{c} \;d{'}_{total}=\frac{n\cdot d{'}_{one}+d{'}_{ensemble}\cdot \sigma_{ensemble}}{\sqrt{n+\sigma_{ensemble}^{2}+2cov\left(S_{optsum},S_{ensemble}\right)}},\; \end{array}$$

where *cov*(*S*_optsum_, *S*_ensemble_) is covariation between random variables *S*_optsum_ and *S*_ensemble_ respectively sampled from the distributions of the optimally summed evidence for individual changes, *S*_optsum_ ∼ *N*(µ = *n × d′*_one_, σ = √*n*) and evidence for ensemble summary change, *S*_ensemble_ ∼ *N*(µ = *d′*_ensemble_ × σ_ensemble_, σ = σ_ensemble_). Model 1 (only optimal summation) is a case of this general model where *d′*_ensemble_ = 0 and σ_ensemble_ = 0 and, hence, *cov*(*S*_optsum_, *S*_ensemble_) = 0. Model 2 (optimal summation with ensemble memory and double sampling) assumes non-zero *d′*_ensemble_ and σ_ensemble_ but *cov*(*S*_optsum_, *S*_ensemble_) = 0, because *S*_optsum_ and *S*_ensemble_ are sampled independently. Model 3 (optimal summation with ensemble memory and single sampling) is the same as Model 2 but the covariation term is simply the product of the standard deviations of the two distributions, *cov*(*S*_optsum_, *S*_ensemble_) = √*n ×* σ_ensemble_. In Model 4 (optimal summation with ensemble memory, single sampling and independent memory noise), 0 < *cov*(*S*_optsum_, *S*_ensemble_) < √*n ×* σ_ensemble_ and the noise of the individual item's evidence for changed is decomposed into two components: One component, σ_sampling_, related to the error in feature sampling from presented items and another component, σ_memory_, related to the error accumulated during memory retention, such that √[σ^2^_sampling_ + σ^2^_memory_] = 1. Because sampling error is perfectly correlated for evidence summation and ensemble summary computation and memory errors are independent, the proportion between σ^2^_sampling_ and σ^2^_memory_ determines the magnitude of the *cov*(*S*_optsum_, *S*_ensemble_). Finally, Model 5 is the same as Model 4, but the σ_ensemble_ is explicitly allowed to vary in a range far exceeding 1 (the noise associated with sampling and remembering individual items) because of the additional integration error.

Our analysis might indirectly suggest that, under reasonable assumptions about sampling and various sources of noise, the use of ensemble statistics in addition to integrated evidence from individual items would have a limited effect on performance, even if observers do calculate these statistics. This conclusion seems to be in line with the main message of Harrison et al. (2021) that the role of ensemble memory in change detection is very limited and that performance mostly can be explained by optimal summation based individual memories. Here, we are moving to our third point that conceptually reconsiders the interpretation of optimal summation and its relation to ensemble memory.

### Optimal summation as ensemble-based decision

**Figure 2.**
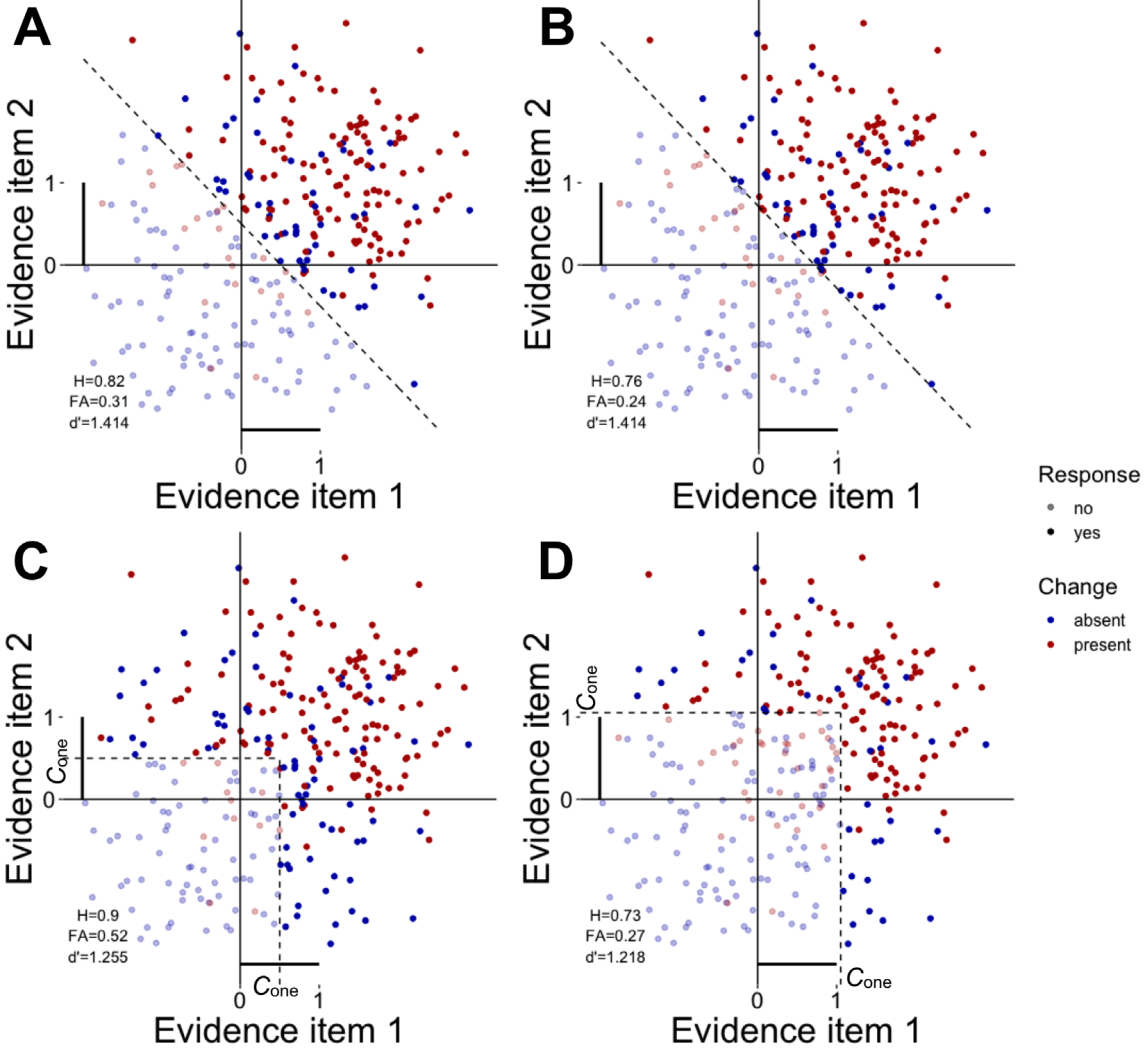
An example model of optimal summation in change detection as a multidimensional signal-detection problem for set size 2 and the one-item change discriminability *d′*_one_ = 1. The same theoretical full-set discriminability (defined as in Equation 3) yields different expected hit rates (H), false alarm rates (FA), and resulting full-set change sensitivity (*d*′) depending on the decision boundary (dashed lines) set by a decision rule and the location of a one-item decision criterion (*C*_one_). (**A**) and (**C**) illustrate situations when the *C*_one_ is set to provide unbiased responses to each of the items alone but this leads to a response bias in the full-set judgements: (**A**) for the optimal decision rule and (**C**) for the minimum decision rule. (**B**) and (**D**) illustrate situations when the C_one_ is set to provide unbiased responses in the full-set judgments: (**B**) for the optimal decision rule and (**D**) for the minimum decision rule.

Although Harrison et al. (2021) define ensemble memory as an extra source of information in addition to the optimal summation of individual change signals, we suggest that optimal summation itself can be considered a form of ensemble-based change detection. According to multidimensional SDT (Hautus et al., 2022), optimal summation is just one of the possible strategies of decision making about multidimensional stimuli (multi-item displays in our case). This strategy implies that evidence from all items is integrated first and then gets compared against a single critical value, *criterion*. That is, on any individual trial, a random number is independently produced for each item from either the signal distribution (change present, mean = *d′*_one_) or the noise distribution (change absent, mean = 0) and then these number are summed up to provide the cumulative evidence for change. This cumulative evidence is then compared against the criterion to deliver a “yes” or a “no” answer. Figures 2A and 2B visualizes this decision rule as a diagonal boundary *C* in an example two-dimensional space (corresponding to change detection in memory set size 2, although this logic can be extended to any other dimensionalities and set sizes). The boundary *C* is a locus of the criterion because the sum of coordinates at every point of this diagonal is the same. Each dot in the space is evidence for a change obtained in a single trial; dot coordinates correspond to the amount of evidence for change obtained from each of the items.

We can see, therefore, that optimal summation is a post-integration decision strategy. As such, we suggest that optimal summation inherently involves ensemble-based decision. By ensemble-based decision, we mean a decision based on evidence coming from all the items together rather than on separate evidence evaluation for one or more items. Importantly, sometimes the cumulative evidence can exceed the criterion even if individual pieces of evidence are below the criterion. In other words, optimal summation suggests that the observer can report a change in the whole display even though no single item brings sufficient evidence for that change. Introspectively, in such situations observers are aware of some change between the sample and test displays but have no idea which particular item or items have changed (Alvarez & Oliva, 2009; Haberman & Whitney, 2011; Ward, Bear, & Scholl, 2016). Note, the ability to discriminate sets based on integrated impression along with the failed discrimination of individual items is one of the cornerstone aspects of ensemble processing that many authors emphasize (e.g., Alvarez, 2011; Ariely, 2001; Corbett & Oriet, 2011; Corbett et al., 2023; Khayat & Hochstein, 2018; Parkes, Lund, Angelucci, Solomon, & Morgan, 2001; Ward et al., 2016; Whitney & Yamanashi Leib, 2018). Of course, optimal summation as a decision strategy based on cue integration is broader than ensemble perception (for example, it can apply to detection and discrimination of multimodal stimuli or multi-feature objects). In the present context, we talk about optimal summation as ensemble-based decision only as long as the change task involves integration of information from multiple objects and across the same feature dimension (color hue, orientation, etc.).

If we define optimal summation as an ensemble-based decision strategy then how can a strategy based only on individual items be defined? In our view, this should be a strategy when the observer keeps track of each item in its pre-change and post-change states separately from other items (“I remember this particular dot had one color at the beginning and now its color is different”). In terms of multidimensional SDT, it means that the observer estimates evidence for change independently along each of the evidence axes which, in turn, implies setting a separate criterion on each axis. The observer decides that an item has changed if evidence for its change exceeds the criterion along a corresponding axis. The whole decision space, therefore, will look like in Figure 2C with a compound decision boundary including two borderlines perpendicular to the axes. Given the nature of the full-set task (all items either change or stay the same), one reasonable strategy do this task is to use the so-called *minimum rule* (Hautus et al., 2022). That is, if there is enough evidence for at least one item changing the observer can answer “yes”; otherwise they answer “no”. Figures 2C–2D illustrate this rule, such that any point to the right from the vertical borderline or above the horizontal borderline warrants a “yes” answer and is shown with more saturated colors. Of course, the minimum rule is an extreme model and different observers can use more conservative rules (say, at least two or three items should provide enough evidence for change to warrant a “yes” answer). However, as shown in Hautus et al. (2022), even if the observer uses the most conservative, maximum rule (all items have to show enough evidence for change) that has a tiny effect on the overall *d′* (*d′*_total_) compared to the minimum rule. What is most important, Hautus et al. (2022) show that any strategy based on the separate criteria for each individual item (as in Figures 2C, 2D) would predict a substantially lower performance than the level of optimum summation (Figures 2A, 2B).

This important consequence of using the two different decision rules, that they lead to different quantitative predictions about the *d′*_total_ based on the same *d′*_one_, provides a way to distinguish between these decision rules. Because the decision space is linearly separable by the criterion in the optimal decision rule (Figures 2A–B), hit (*H*_total_) and false alarm (*FA*_total_) rates can therefore be defined as normal cumulative density functions Φ of distances between the centers of the corresponding multivariate distributions (which is exactly *d′*_total_) and the criterion (Hautus et al., 2022):

(6) $$\begin{array}{c} H_{\mathrm{total}}=\Phi \left(d{'}_{\mathrm{total}}-C\right),\; \end{array}$$

(7) $$\begin{array}{c} FA_{\mathrm{total}}=\Phi \left(-C\right).\; \end{array}$$

Substituting these expressions into Equation 1 leads simply to the *d′*_total_ in the result. Therefore the optimal decision rule is indeed a strategy that provides performance at the level of optimal summation.

In the minimum rule-based decisions, the total probabilities of hits and false alarms include, therefore, all cases when either of the items produces a hit or a false alarm. This can be viewed as a complement to the probability that none of the items produces a hit or a false alarm. These latter probabilities can be calculated as products of one-item misses (1-*H_i_*) or correct rejections in (1-*FA_i_*) that, in turn, depend on individual *d′* (*d′_i_*) and individual criteria (*C_i_*) as follows:

(8) $$\begin{array}{c} \;H_{total}=1-\prod_{i=1}^{n}\left(1-H_{i}\right)=1-\prod_{i=1}^{n}\left[\Phi \left(-d{'}_{i}+C_{i}\right)\right],\; \end{array}$$

(9) $$\begin{array}{c} \;FA_{total}=1-\prod_{i=1}^{n}\left(1-FA_{i}\right)=1-\prod_{i=1}^{n}\left[\Phi \left(C_{i}\right)\right],\; \end{array}$$

where *n* is the memory set size and *i* is the individual item's number. Consequently, if all items have the same one-item *d′* (*d′_one_*) and an observer uses the same criterion for all items (*C_one_*), then the *H*_total_ and the *FA*_total_ are simply:

(10) $$\begin{array}{c} H_{\mathrm{total}}=1-{\left[\Phi \left(-d{'}_{\mathrm{one}}+C_{\mathrm{one}}\right)\right]}^{n},\; \end{array}$$

(11) $$\begin{array}{c} FA_{\mathrm{total}}=1-{\left[\Phi \left(C_{\mathrm{one}}\right)\right]}^{n}.\; \end{array}$$

Substituting these expressions to Equation 1 yields lower predictions for the *d′*_total_ as a function of the *d′*_one_ than the optimal rule model (Equation 3). The difference between the *d′*_total_ predicted by the minimum rule model and that predicted by the optimal rule model increases with memory set size. For example, assuming an observer using an unbiased decision criterion from the one-item condition (that is, a criterion that allows to keep the proportion of “yes” answers in that condition at about 0.5), in set size 2, the *d′*_total_ equals ∼1.41*d′*_one_ for the optimal rule model and ∼1.25*d′*_one_ for the minimum rule model; in set size 4, these are 2*d′*_one_ and ∼1.62*d′*_one_, respectively; in set size 6, these are ∼2.45*d′*_one_ and ∼1.9*d′*_one_, respectively. However, as Figures 2A and 2C show, keeping the unbiased one-item criterion in the full-set condition should substantially increase the overall proportion of “yes” answers. Both the optimal rule and the minimum rule models require the observer to raise their criterion from the one-item level to stay unbiased in the full-set condition (Figures 2B and 2D). As can be seen from Equations 1, 6, and 7, this adjustment of criterion does not change the empirically estimated *d′*_total_ if an observer uses the optimal decision rule. However, in the minimum rule model, raising the decision criterion for every single item further decreases the empirically estimated *d′*_total_ (whereas the theoretical *d′*_total_ as a distance between the multidimensional signal and noise distributions stays the same). For example, in set size 2, the minimum rule model with an unbiased full-set criterion predicts that the *d′*_total_ equals ∼1.22*d′*_one_; in set size 4, it predicts that the *d′*_total_ equals ∼1.44*d′*_one_, in set size 6, it predicts that the *d′*_total_ equals ∼1.57*d′*_one_.

To sum up, Harrison et al. (2021)’s conclusions about ensemble-free change detection are mostly based on demonstrations that their observed *d′*_total_ did not exceed the predictions based on the optimal rule model. However, under the minimum rule model that we consider better fitting the strict definition of item-based decisions, the predictions for the *d′*_total_ are substantially lower and we will further show that Harrison et al.’s data actually exceed these predictions, suggesting that some decisions could be in fact ensemble based.

### Our study

Our study consists of two parts. In the first part, we have run two experiments addressing the assumption that one-item *d′* reflect the “ensemble-free” mode of change detection. In these experiments, we manipulated the range of feature variation, which is related to the precision of ensemble statistics that can be extracted from a set (Dakin, 2001; Fouriezos, Rubenfeld, & Capstick, 2008; Im & Halberda, 2013; Morgan, Chubb, & Solomon, 2008; Solomon, 2010; Marchant, Simons, & de Fockert, 2013; Rosenholtz, 2001; Utochkin & Brady, 2020; Utochkin & Tiurina, 2014; Watamaniuk & Sekuler, 1989). This range manipulation is directly linked to the aforementioned notion about item similarity as a determinant of items being perceived as a part of the same ensemble or not (Utochkin, 2015). In the second part, we implemented the two possible decision models (optimal rule and minimum rule) to demonstrate how the same estimated one-item *d′*-s can yield substantially different predictions on the upper-bound *d′*_total_. We then compared the data (from both Harrison et al., 2021, and our Experiment 1) with the model predictions to answer the question whether these data show evidence against an ensemble component in change detection.

## Experiment 1

### Participants

Twenty-one students of the HSE University (three males, 18 females, mean age = 18.76, age *SD* = 0.7) took part in the experiment for course credits. All participants were tested having normal color vision and normal or corrected-to-normal visual acuity and reported having no neurological problems. Before the experiment, all participants gave informed consent. Harrison et al. (2021) showed that 20 participants is a sufficient sample size to obtain conclusive Bayes factors in a design like ours (using Bayesian statistics). Therefore our sample sizes were informed by this estimate.

### Stimuli and procedure

Stimuli were presented on a standard cathode-ray tube monitor with a refresh frequency of 75 Hz and a 1600 × 1200-pixel spatial resolution. The monitors were calibrated using a color calibration device Spyder 5 ELITE. The experiment was run on PsychoPy 3 for Linux (Peirce et al., 2019). The viewing distance was about 50 cm. From that distance, one pixel subtended approximately 0.032° of visual angle.

Stimuli were outlined colored discs (∼2.4° in diameter) located on an imaginary circumference with a radius of ∼4.8° from a fixation point on a uniform gray background. The absolute stimulus positions on the imaginary circumference were randomized between trials but the distance between any two discs was always 90° of arc. Filler colors of the discs were chosen from the CIE Lab color wheel (L = 70, a = 20, b = 38, radius = 60, as in Brady & Alvarez, 2015a).

**Figure 3.**
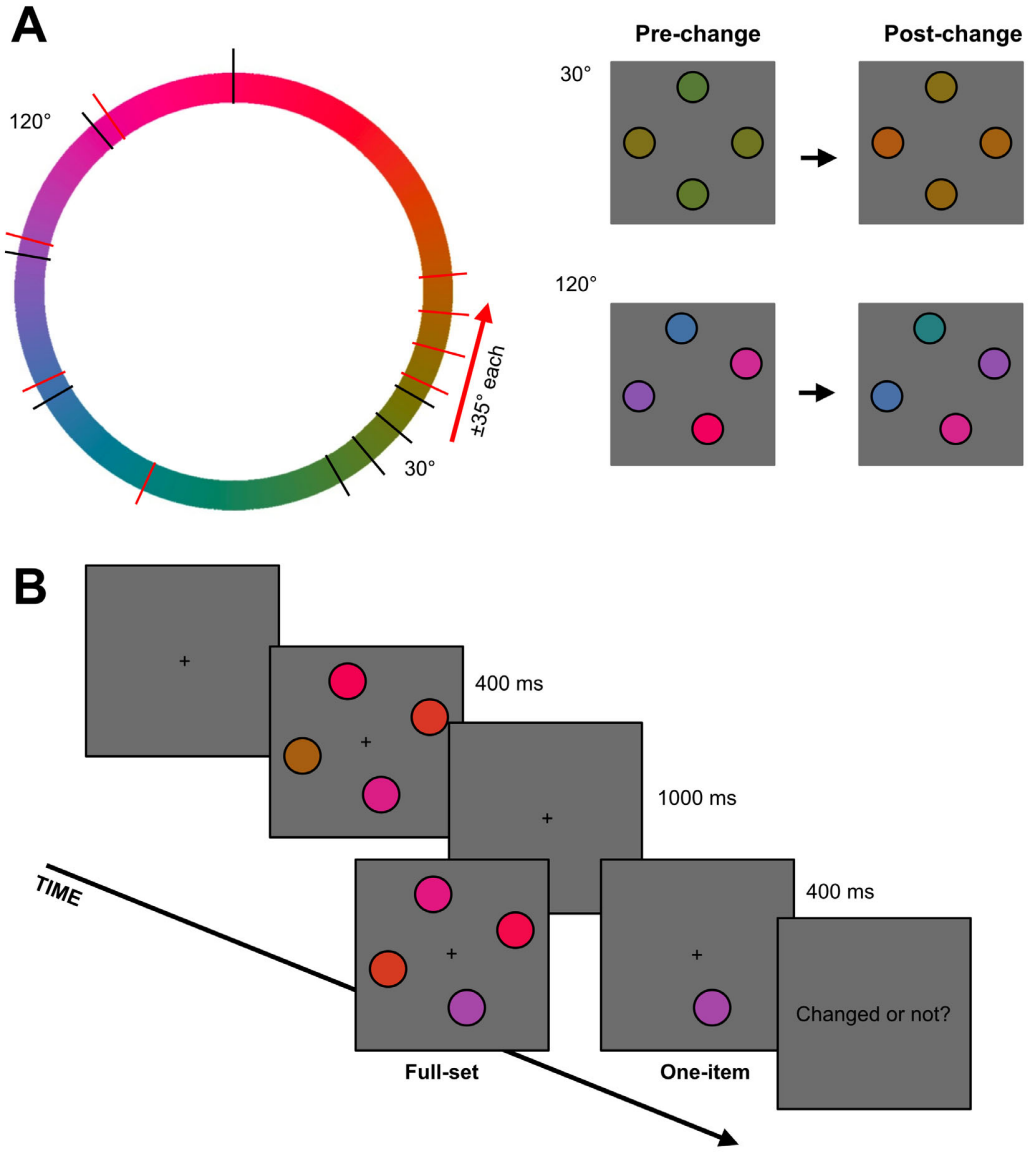
Stimuli and procedure of Experiment 1: (**A**) example low-range and high-range color distributions of pre-change (black marks on the color wheel) and post-change (red marks) stimuli; (**B**) the time course of a typical trial.

Memory sets always consisted of four discs of different colors. These colors could have either the narrow-range (30°) or the broad-range (120°) distribution along the color wheel (Figure 3A, left). To set a color distribution on each particular trial, we first picked a random angle from the color wheel (from 1° to 360°, step 1°), which served as a center (mean) of the distribution for that trial. In the narrow-range distributions, individual colors were −15°, −5°, 5°, and 15°. In the broad-range distributions, the colors were −60°, −20°, 20°, and 60° from the mean. Test displays contained either four discs presented at the same locations as in the original memory set (full-set condition), or one disc presented at one of the locations from the memory set (one-item condition) chosen at random. In no-change trials, the colors of the items of the test set were the same as those of the memory set (each preserving its original location). In change trials, all colors of the full-set test displays differed by the same value (35°) and in the same direction (clockwise or counterclockwise) along the color wheel from the memory colors at respective locations (Figure 3A). This manipulation shifted the mean color in the full-set condition, as in the mean change condition of Harrison et al. (2021) experiments. In the one-item condition, the color of a single presented item also shifted 35° in either direction in the case of a change.

Each trial (Figure 3B) started with a white fixation cross presented at a screen center and maintained until the end of the trial. 500 ms after the fixation onset, a memory display with four color discs was presented for 400 ms. The memory display was followed by a blank retention interval for 1000 ms when the memory set disappeared from the screen. After the retention interval, a test display was presented for 400 ms. Participants had to respond whether they had detected a change by pressing an “L” button for a “yes” response or an “A” button for a “no” response. A feedback followed a button press for 500 ms informing the participants whether their response had been correct or incorrect.

The experiment consisted of 400 trials that were divided between the conditions as follows: 2 post-test display types (one-item vs. full set) × 2 ranges (narrow vs. broad) × 2 change presence conditions (present vs. absent) × 50 repetitions. The serial order of trials was fully randomized both across and within participants. The sequence of trials was divided into four blocks of 100 trials in each. Participants could rest between the blocks. At the beginning of the experiment, a short block of 32 practice trials was run (the data from this block were not analyzed).

### Analysis

Proportions of “yes” responses were calculated for change-present (hit [H]) and change-absent (false alarms [FA]) trials within each condition and each participant. These proportions were then used to calculate the *d′*, as in Equation 1. To deal with hit rates of 100% (which makes a *z*-score undefined), we applied a correction of both hit and false rates suggested by Hautus (1995). Although only one participant showed 100% hits in one condition, this correction was applied to all the data for uniformity.

As in Harrison et al. (2021), we used Bayesian *t*-tests with a default prior (Rouder, Speckman, Sun, Morey, & Iverson, 2009). Harrison et al. (2021) used these tests to compare observed *d′*_total_ against those predicted by the optimal summation model given observed *d′*_one_. We will address this comparison later, when we test our and Harrison et al. (2021)’s data against the two decision models (optimal summation and minimum rule). Here, we focus on estimating the effects of color range of a memory set on change detection. In particular, we ask whether this effect is existent in one-item detection and in full-set detection. Therefore our critical comparisons are between the narrow-range *d′* and the broad-range *d′* within the full-set and one-item conditions. The Bayesian t-tests were performed using the package “BayesFactor” for R (Morey, 2018).

### Results and discussion

**Figure 4.**
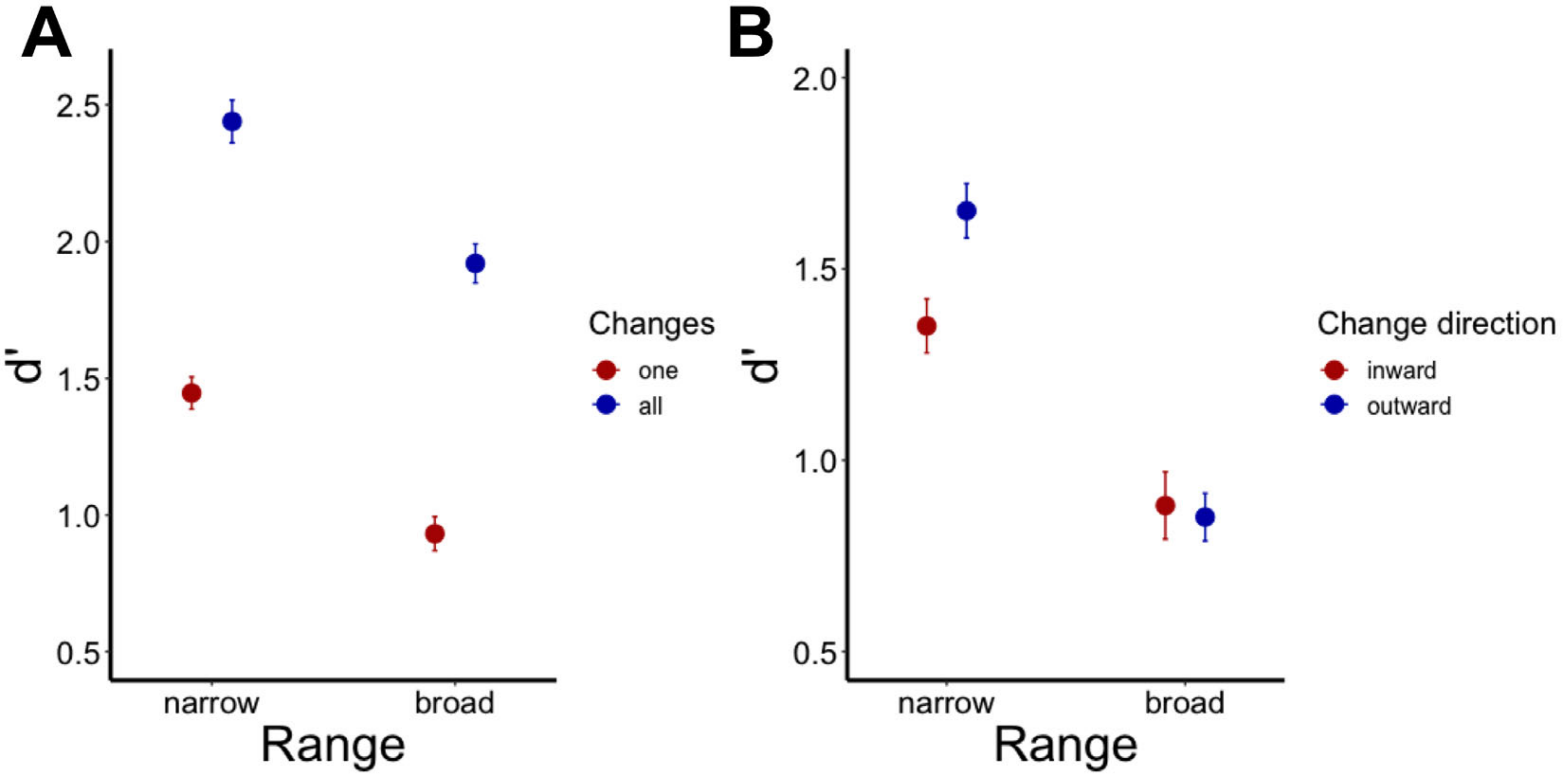
Change-detection performance (*d′*) (**A**) as a function of range and the number of post-change items in Experiment 1 and (**B**) as a function of change direction and range in Experiment 2. Error bars depict ±1 within-subject standard error of the mean.

We found strong evidence that our observers were substantially better at change detection when memory sets were narrow-range compared to broad-range (Figure 4A). Importantly, this was the case both for the one-item condition (*BF*_10_ = 271.9) and for the full-set condition (*BF*_10_ = 29.5). These findings suggest that the distributional properties of the whole memory set and not only individual item discriminability contribute to change detection. In both one-item and full-set trials, it is easier to detect a change if the original memory set consists of highly similar items, as in our narrow-range condition. This can be interpreted in terms of signal-to-noise ratio, when the observer evaluates the amount of change across displays (signal) in relation to feature variability within the display (noise). Roughly, the observer evaluates how much the colors differ between the displays compared to how much they differ within the display. In theory, this strategy can be implemented without computing summary statistics: The observer can estimate pairwise differences between some of the pre-change items and then decide whether the post-change difference or differences are bigger than these original differences in the initial displays. Therefore, if color heterogeneity is small, this signal-to-noise ratio should be larger predicting better performance. When heterogeneity increases, then the signal-to-noise ratio decreases predicting a loss in performance. Alternatively, the advantage of the narrow-range sets can be interpreted as the availability of ensemble information to combine with information about the individuals, as was suggested in the introduction: For example, the impression of the “mean” color is stronger when items are highly similar and it can be easier to compare a post-change impression with a pre-change one both in terms of individual change and change relative to the mean. Whatsoever, any possible interpretation should acknowledge the fact that change detection performance strongly depends on the feature distribution of the memory set, even if it is tested on a single item.

## Experiment 2

Although we found the advantage of the narrow-range displays in the one-item condition of Experiment 1, this result can be potentially interpreted without a reference to ensemble representation. For example, it can be possible that observers check post-change colors for their presence or absence in the pre-change set. This involves pairwise comparisons between all post-change colors (one or four, depending on condition) against all pre-change colors without any integration (“Are there any color(s) that I have not seen in the first display?”). We will refer to this strategy as “novelty detection” account. Note that this strategy implies that observers do not keep track of spatial sources of memoranda, that is, they compare a post-change color in a given location with pre-change colors in all locations. Alternatively, if observers still keep track of the spatial sources they can nevertheless commit *swap* errors when they occasionally compare a given post-change color with a pre-change color sampled from a wrong location. We will refer to this scenario as “swap” account.

In theory, both these strategies could give rise to the narrow-range advantage observed in Experiment 1. Given that the overall range of the narrow-range displays was 30° and the target change was 35°, this change was basically in the direction *away* from all four pre-change colors (it can be seen in the in Figure 3A, left panel, where the narrow distributions of pre-change (black marks) and post-change (red marks) colors do not overlap on the color wheel). In contrast, our broad-range displays had a 120° range, in which case the 35° step of change pushed the post-change item away from the target but it could pull it toward some non-targets at the same time (in the same example in Figure 3A, where the broad distributions of pre-change (black marks) and post-change (red marks) strongly overlap on the color wheel). Specifically, the only possibility for the post-change item to move away from all pre-change colors was when the target was an extreme color in a pre-change color distribution and the change was outside this distribution (25% chance); in all other cases, the post-change color got closer to at least one of the non-target colors. In other words, changed colors in the broad-range displays on average were more similar to the pre-change colors than in the narrow-range displays. This could result in poorer discrimination of novel colors as well as in the increased number of swap errors (e.g., Oberauer & Lin, 2017). In Experiment 2, we balanced occurrences of target changes toward or away from the whole color distribution which allowed us to better control similarities between the post-change target color and pre-change non-target colors. With this control, we could test whether this “novel-color” or “swap” scenario could account for the narrow distribution advantage from Experiment 1.

### Participants

Twenty-two students of the HSE University (seven males and 15 females, mean age = 18.9, age *SD* = 0.45) took part in the experiment. All were tested having normal color vision and normal or corrected-to-normal visual acuity and reported having no neurological problems. At the beginning of the experiment, the participants gave written informed consent. Two participants were not included in the analysis because of low overall performance (less than 60% correct answers).

### Stimuli and procedure

**Figure 5.**
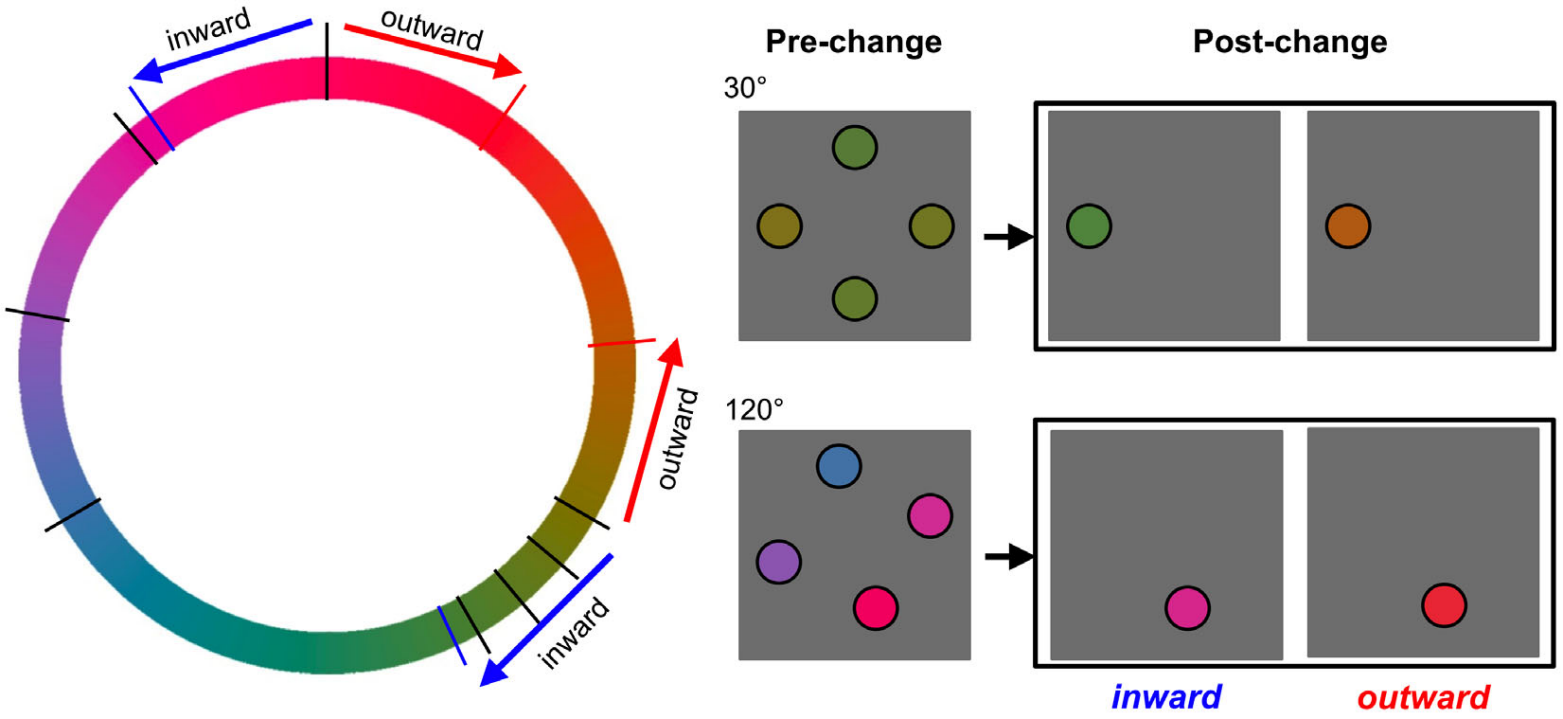
An illustration of the inward (blue marks on the color wheel) and outward (red marks) changes of a post-change item relative to the pre-change distribution (black marks) in Experiment 2.

We used the same apparatus and software as described for Experiment 1. Stimuli and procedure were also similar to Experiment 1 in terms of trial time course, memory set size, color ranges of memory sets, and the change magnitude. Two critical changes were made to the procedure. First, we tested only the one-item post-change condition. Second, we systematically manipulated the direction of change relative to the color distribution (Figure 5). In this experiment, only one of the extreme colors from the memory set could change (that is, either the most clockwise or the most counterclockwise member of the color distribution). The change could be either “inward” or “outward.” In the “inward” case, the direction of a change was toward the rest of the colors. For example, if a pre-change item in a memory set had the most clockwise color in the original distribution, then its post-change version was 35° more counterclockwise moving closer to the rest of the items. In the “outward” case, the direction of a change was away from all of the pre-change colors. As can be seen, inward changes make the test item most similar to one of the non-target items from the memory set. Interestingly, the difference between the test item and the most similar non-target is always 5°, even though intercolor distances differed between the narrow and broad color ranges. Hence, the “inward” change, when present, was always a color almost identical to one of the pre-change colors, both in the narrow-range and broad-range conditions. Moreover, the average difference between the post-change target color and all non-target colors remained the same as between the pre-change color and the non-target colors: Increased differences from some of the pre-change items were compensated by decreased differences from others. In contrast, the outward changes make the post-test item dissimilar from any of the colors. So, the inward changes always cause a replacement of a target with another item from the original memory set, whereas the outward changes always bring a new color into a test display. Therefore, if observers compare a test item with a list of four independent individual colors in their memory, then their sensitivity to outward changes should be greater but it should not depend on whether the entire distribution is broad or narrow. Moreover, because inward and outward changes are now balanced between the ranges, the sensitivity should be the same for the narrow- and broad-range displays.

### Results and discussion

First, we found strong evidence for the range effect on overall performance (*BF*_10_ = 16.7 × 10^4^), suggesting that participants were better at detecting item changes if the original memory set was narrow-range (Figure 4B). Second, we found some evidence that the outward changes were detected better than the inward changes (*BF*_10_ = 2.85) in the narrow-range color distributions. We also found evidence against a difference between the outward and inward changes in the broad-range stimulus distributions (*BF*_10_ = 0.238).

In Experiment 2, we replicated the finding from Experiment 1 that the narrow-range memory sets yielded better change detection than the broad-range ones. This result could not be expected if the observers had compared pre-change and post-change items only at the target location with no reference to other colors. Further, if the observers had compared a post-change color with all individual pre-change colors to estimate its novelty or if they occasionally made swap errors, we would not have expected the differential effect of range on sensitivity because inward and outward changes were balanced across ranges in Experiment 2. An additional argument against the “novelty detection” account is that the *d*′ in the inward condition were substantially above 0. Indeed, if the observers simply checked all pre-change colors, then they could not discriminate between no-change trials (the post-change color is the same as the pre-change color at the same location) and change trials (the post-change color is almost the same as one of the pre-change colors at a different location). In sum, the pattern of results in Experiment 2 suggests that the range effect on the *d′*_one_ is provided by the combination of information from the target item with respect to its location and some integral distributional information that we broadly can refer to as ensemble information.

In Experiment 2, we found no evidence for the direction of change effect on performance in the broad-range condition. This observation is in line with a change-detection mode when the observer mostly relies on changes to individual items at their locations and does not strongly relies on ensemble cues. However, the direction of change is more important for the narrow range suggesting that observers take into account the change relative to the whole distribution. We can conclude, therefore, that the distributional properties of the whole set (that we can call ensemble properties) play a greater role in change detection if the set consists of relatively similar items. On the other hand, when the items in a set get more dissimilar, observers can rely on memories of individual items to a greater extent. This conclusion is partly consistent with the results of exploratory analyses by Harrison et al. (2021). Note that that reliance on items in the broad-range condition or on ensemble in the narrow-range does not necessarily imply two distinct modes or strategies of change detection. Observers can actually rely on both sources of information but the effectiveness of ensemble contribution can depend on its reliability.

### Testing the data against optimum rule and the minimum rule

To test the data against these two decision models, we performed a Bayesian meta-analysis similar to that performed by Harrison et al. (2021) for their experiments. For that meta-analysis, we took the data from all experiments by Harrison et al. (2021) where memory set size was 4 (Experiments 3–6). Following Harrison et al. (2021) design and analyses, two conditions of their experiments (mean change and variance change in the full-set trials) were represented as different data points. We added the data from our Experiment 1 where both *d′*_one_ and *d′*_total_ were directly measured. We included the narrow-range and the broad-range conditions as separate data points. For each observer, we calculated two predicted *d′*_total_ based on their *d′*_one_. One predicted *d′*_total_ was based on the optimal summation model (Equation 3) that is equivalent to the prediction from the optimal decision rule (Equations 6 and 7). Another predicted *d′*_total_ was based on the minimum rule model (Equations 10 and 11). To remind, for the minimum rule model, the predicted *d′*_total_ depends not only on the *d′*_one_ but also on the decision criterion set on evidence obtained from each item (*C*_one_). Therefore, for each participant from our Experiment 1 we used a grid search to fit a *C*_one_ whose substitution into Equations 10 and 11 provided the same Yes rate (average of *H*_total_ and *FA*_total_) as that observed in this participant. The best-fit pair of *H*_total_ and *FA*_total_ from Equations 10 and 11 were then substituted into Equation 1 to find a minimum-rule prediction for the *d′*_total_. For Harrison et al. (2021) data set, where only *d′*_one_ is available without any information about proportions of hits and false alarms, we simply assumed an unbiased response strategy for all observers and, hence, fit their *C*_one_ to Yes rate = 0.5.

**Figure 6.**
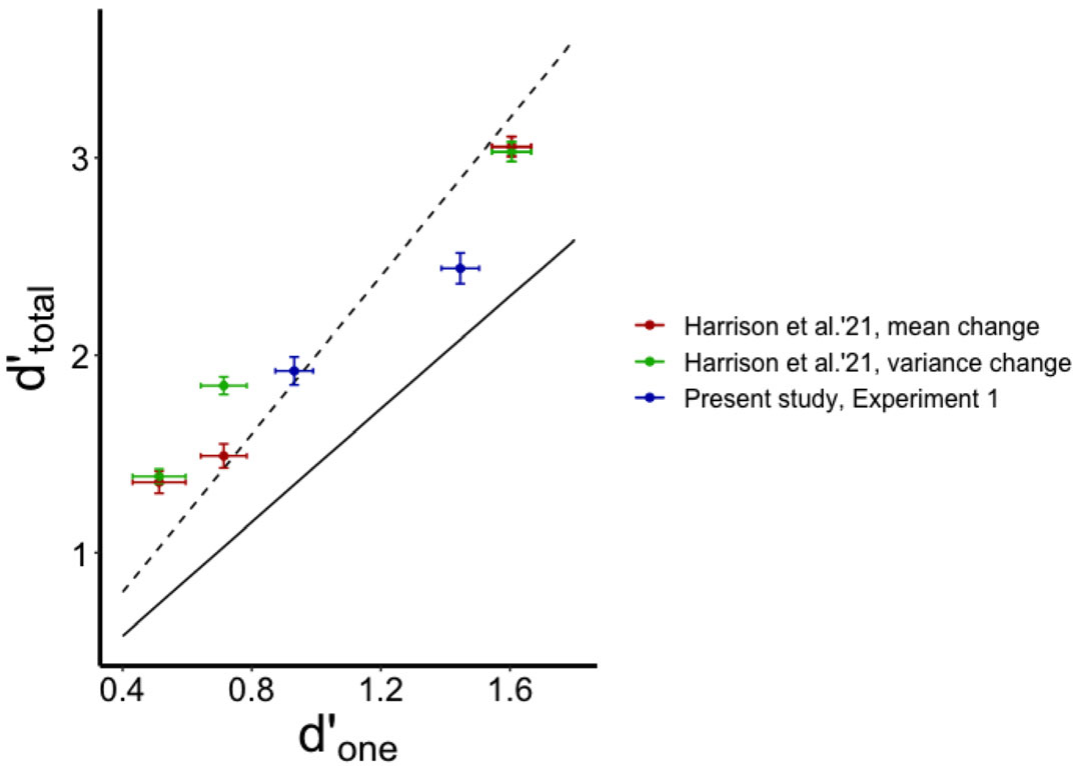
Observed *d′*_total_ (from data reported by Harrison et al., 2021 and our own data) against the predictions based on the optimal rule and the minimum rule models. Note: The prediction line for the minimum rule model (solid line) is set for unbiased full-set responses (Yes rate = 0.5) that we assumed for the data from Harrison et al. (2021). For our own data (Experiment 1), the average Yes rate = 0.45, but the current figure does not show this prediction line because its slope is just negligibly lower than the slope of the shown prediction line. Error bars depict ±1 within-subject standard error of the mean.

For each condition of each experiment, we compared the observed *d′*_total_ against the predictions from the optimal rule model and from the minimum rule model. As in Harrison et al. (2021), the null hypothesis was that the observed *d′*_total_ are not greater than the model predictions. Then we calculated a meta-analytic Bayes factor (Morey, 2018; Rouder & Morey, 2011) to evaluate evidence for or against the null hypothesis across the experiments. We found that most of the data supported weak to moderate evidence for the null hypothesis with respect to the optimal rule model (0.07 < *BF*_10_ < 0.41; exceptions included the variance-change conditions of Experiments 5 and 6 and the mean-change conditions of Experiment 6 from Harrison et al., 2021: *BF*_10_ > 5.4). The meta-analysis showed weak overall evidence for the null hypothesis (*BF*_10_ = 0.52). On the other hand, we found strong evidence against the null hypothesis for all data points with respect to the minimum rule model (*BF*_10_ > 38). The meta-analysis showed very strong evidence against the null hypothesis (*BF*_10_ = 4.4 × 10^38^). We can conclude from these analyses that, in most cases, the observers performed no better than a model observer using the optimal decision rule (this is basically consistent with Harrison et al., 2021) but outperformed a model observer using the minimum rule decision rule. This is illustrated in Figure 6, where the data points are mostly concentrated around the prediction line of the optimal rule and, at the same time, they are fairly above the prediction line of the minimum rule.

The results of the meta-analysis of data by Harrison et al. (2021) with two additional data points from our own study support Harrison et al.’s conclusion that observers basically accept the optimal strategy of change detection based on the sum of evidence picked from all items. As we noted in the introduction, the optimal rule implies that some decisions can be based only on a general impression of the overall change without detecting any item change. We can define such decisions as mostly ensemble-based. Such decisions provide the predicted advantage for sensitivity in the optimal rule strategy compared to the minimum rule strategy, when at least one item has to provide a change signal strong enough to exceed the decision boundary. Hence, we conclude that in the full-set trials observers rely on both individual items and ensemble information.

Although most of the data points in Figure 6 are indeed close to the optimal-summation level, there is one interesting exception, namely the data point for the narrow-range condition from our Experiment 1. In this condition, observers showed performance well above the minimum-rule level but also below the optimal level. It suggests that observers rely on some evidence integration that is, nevertheless, not perfect. There can be many possible explanations for why the integration efficiency falls short of the optimal level in this condition. However, modeling these explanations would require introducing more complicated algorithms and parameters. For example, observers can directly compare noisy average signals between the displays in the full-set trials (in the one-item trials, the average post-change signal is simply a sample from a one-item distribution). Such a model would predict the full-set advantage and the amount of this advantage is determined by two parameters, mean color discriminability and individual-from-mean discriminability. Because neither we nor Harrison et al. (2021) have measured these parameters, no precise predictions can be made at this point. We should note that we do not suggest that our observers fully relied solely on this averaging strategy in the narrow-range condition. If this was the case, we would expect *d′* = 0 in the “inward narrow” condition of Experiment 2 where the target color shifted from one tail of the color distribution to another but its distance from the mean did not change. It could be a more complex mixture of different strategies in different trials that eventually resulted in the suboptimal performance level compared to the pure optimal-rule model. However, a failure to perform optimally does not automatically imply that observers did not use integrated, ensemble-based information.

Another possible explanation of the suboptimal performance in the narrow-range condition of Experiment 1 can be considerable noise correlation between the samples. This explanation goes beyond the assumptions of the optimal summation model, as it is presented in our Equations 1 through 4 and in Harrison et al. (2021), where each individual piece of evidence is put orthogonal to the rest. However, this correlation is plausible given evidence for correlated noise in neural populations coding similar features (Averbeck, Latham, & Pouget, 2006). That is, in the narrow-range condition where colors are similar, a positive correlation between individual sampled colors can emerge (even if these colors are sampled independently) and that overall decreases the benefit from their integration (Hautus et al., 2022; Wixted, Vul, Mickes, & Wilson, 2018). Formally, the detrimental effect of individual noise correlation on the summation benefit can be most easily shown by adding the covariance term (same as in Equation 5) to the denominator of the optimal summation equation (Equation 2b). If the covariance parameter is non-zero, it is clear that the *d′* should become smaller.

## General discussion

Our study demonstrated that change detection performance depends not only on the discriminability between pre-change and post-change items but also on between-item relationships that we refer to as ensemble properties. We considered two arguments. First, we found evidence for the ensemble effect even in the one-item change condition that was assumed to represent the baseline for the “ensemble-free” mode of change detection, according to Harrison et al. (2021). Therefore we showed that observer's performance in the one-item condition should not always be taken as a default measure of the ensemble-free mode of visual working memory. Second, although performance in the full-set condition rarely exceeded the level of optimal summation of individual change signals, summation itself implies that some decisions are based only on the sum of evidence rather than on any of the individual items (Allik, Toom, Raidvee, Averin, & Kreegipuu, 2013; Haberman & Whitney, 2011), at least if the optimal decision rule is used. Our demonstration is in line with the previous evidence for the role of ensemble statistics in working memory tasks (Brady & Alvarez, 2011; Brady & Alvarez, 2015a; Brady & Alvarez, 2015b; Corbett, 2017; Utochkin & Brady, 2020).

### What is an ensemble representation used in visual working memory?

Although we view optimal summation as a form of post-integration, ensemble-based decision, we consider Harrison et al. (2021) goal to distinguish between mere evidence integration and ensemble statistics used on top of that very important for understanding the true nature of representations that people use in change detection or other working memory tasks. However, in our view, the current version of the model is not sufficiently specific to unambiguously capture these different representations. The fact that observers perform at the optimal level suggests that they do integrate information about multiple items before making a decision. However, as we have shown above, many various properties of representational noise associated with sampling, integration, memory retention can influence the effectiveness of evidence summation and ensemble statistics at the same time. Because of that, a clear quantitative prediction for what pure “no-ensemble” memory and how ensemble statistics should alter performance is complicated. The model also does not specify how observers showing the optimal level of performance integrate information computationally. It can be a simple sum of evidence for change collected from all items, as Harrison et al. (2021) suggest. Alternatively, for example, observers can estimate *z*-distances between the average pre-change feature and the individual (in the one-item condition) or the average (in the full-set condition) post-change feature, which involves the representation of summary statistics. Many other ways of integration can be considered in other models and experimental designs in the future. (Beyond the main scope of the present article, multiple previous studies have concluded that ensemble integration may be actually suboptimal. This suggests that the effective contribution of individual items into the ensemble representation can be unequal (Dakin, 2001).)

Having said that, we do not mean that observers performing at the level of optimal summation directly rely only on ensemble statistics, especially when half of all trials are one-item trials. It is possible that they try to track both individual changes and changes in ensemble statistics. Because cumulative evidence is a sum of individual pieces of evidence, there should be a trial-by-trial correlation between item-based and ensemble-based impressions. For example, if cumulative evidence is strong enough to exceed the criterion, then it is likely that evidence from at least one item is also strong enough to exceed the criterion: That is, in many trials the observer will be able the detect changes both in at least one individual item and in the whole set (Oriet, Giesinger, & Stewart, 2020). It is clear, however, that the correlation between cumulative evidence and each individual piece of evidence should decrease with growing memory set size. That is in line with an intuitive idea that, as set size grows, the optimal observer should rely on the integrated ensemble impression more than on individual items.

The idea that observers benefit from combining information based on their integrated impression (be it sum of individual signals or a summary statistic) and individual items have strong parallels in signal-detection models of recognition memory in n-alternative choice tasks, such as suspect identification in police lineups (Wixted et al., 2018). In many such models, the observer chooses an item producing the strongest memory-match signal (which should be replaced by the memory-mismatch signal in the case of change detection) but only if the integrated signal exceeds a certain criterion. Such models can be used for a further development of SDT models of change detection, especially given that most of the change detection experiments reported in the literature involve changing only one item, even if the whole set is presented after the retention interval. Noteworthy, the standard models of lineup memory do not imply any ensemble information presented at encoding (simply because the lineup identification procedure implies that only one, if any, of the presented persons could be encoded at the crime scene). In contrast, the change detection task often requires to encode several items. Therefore the models of lineup recognition should be adjusted accordingly to include the possibility of ensemble representation at encoding. This, in turn, can be a fruitful step for the lineup recognition field itself because the models considering ensemble information at encoding would be able to better account for possible episodic context effects on recognition (for example, the fact that the suspect can be not the only person remembered from the crime scene).

Although we disagree with Harrison et al. (2021) on their conceptual interpretation of optimal summation as non-ensemble decision, we agree with their claim that the contribution of ensemble information to visual working memory is limited. By this, we do not mean that observers just barely rely on ensemble information. Rather, we mean that the availability and reliability of ensemble information itself limits its utilization in working memory tasks. Both color and orientation spaces are very broad and not all randomly picked features from these spaces will form a strong group that can be described with a single set of summary statistics. For example, if all colors are drawn from a relatively narrow range spanning various blue-red shades then an ensemble-based strategy of change detection can be quite useful (“I can see that this new item is substantially redder than the original set on average”). But if all colors are categorically different then this strategy is not that useful because no meaningful average or variance can be coded (if the set included a red, a blue, a green, and a yellow item, then a slight change of one or even all four colors will not affect the general impression of the average “red-blue-green-yellow-ness” because each color is just a different category).

Even if the categorical nature of a relevant feature dimension is not that obvious (as in the case of size or orientation; although see Wolfe, Friedman-Hill, Stewart, & O'Connell, 1992 for their notes on orientation categories), increasing feature heterogeneity dramatically decreases the discrimination of ensemble statistics, be it mean (Dakin, 2001; Im & Halberda, 2013; Gorea, Belkoura, & Solomon, 2014; Maule & Franklin, 2015; Solomon, 2010) or variability (Morgan et al., 2008; Solomon, 2010). That is, exactly the same amount of change in an individual item can be sufficient to cause an impression of ensemble change if the feature distribution is narrow, but insufficient to cause that impression if the feature distribution is broad. In the former case, change detection can be based on both item-based and ensemble-based sources of information, whereas in the latter case it should be mostly item-based. That is what we observed in our Experiment 2. In the narrow-range condition, the *d′* strongly depended on whether the target change was inward or outward, that is, whether it changed its distance from the mean and whether it fell out of the range. In contrast, the direction of change was not important in the wide-range condition, suggesting more of the item-based change detection strategy.

What kind of representational basis can support the plausibility of ensemble representations in visual working memory? It is widely proposed that visual working memory (at least, memory for basic continuous features such as color, orientation, or location) strongly relies on neural encoding mechanisms used for perception (d'Esposito & Postle, 2015). One of the most basic such mechanisms is population coding, that is, the distributed activity of multiple feature selective neurons. It is suggested that the properties of this distributed neural activity, both within a population encoding a single item or a single feature and across populations encoding multiple items or multiple features, can accurately account for many measurable behavioral limitations of visual working memory (Bays, 2014; Bays, 2015; Schneegans & Bays, 2017; Schneegans & Bays, 2018). We suggest that ensemble information can be also represented in a population code that arises from pooling local information from neural populations with small receptive fields by neural populations with large receptive fields within the visual hierarchy. Three recent works proposed various quantitative population-like models that account for ensemble representations and the role of relational information in visual working memory. Utochkin et al. (2023) suggest a pooling and population coding model (akin to earlier pooling models considering related phenomena, e.g., Harrison & Bex, 2015; Webb, Ledgeway, & McGraw, 2010) that captures the variety of ensemble representations, including the computation of average and variability, as well as setting boundary conditions for all items being perceived as belonging to one or several clusters. Robinson and Brady (2023) conceptualize the ensemble representation as a pooled familiarity signal produced by individual items in noisy feature-selective channels (Schurgin, Wixted, & Brady, 2020), an idea having a strong reference to population coding. Udale, Gramm, Husain, & Manohar (2021) suggest that population codes can apply both to absolute values of individual features and to feature relations at different hierarchical levels of processing and that observers rely on a combination of these population responses when they perform a visual working memory task.

It is important to note that the two sets of arguments that we considered in this article (one coming from the SDT analysis of the optimal-summation model and another coming from the experiments) concern two different aspects of the role of ensemble information in change detection. Whereas our model analysis focuses on the way observers make a decision about multiple changes given their ability to spot a change in a single item, our experiments mostly show how the sensitivity to the single item is modulated by the distributional properties of the entire set. At this point of our work, we acknowledge a gap between these two aspects in terms of the possibility of a single formal model. Ideally, this is a model that would capture the integration between individual-level and ensemble-level information in provision of the single-item change detection (determining components of *d′*_one_) and further integration when change detection in multiple items is performed (the job that the current version of the model does). As was said above, the main reason for the lack of such a model is the variety of qualitatively different plausible strategies that can explain the modulatory effect of feature distribution on the *d′*_one_. One of the possible directions for the future model development can be focused on both empirical and computational work to disentangle between these candidate strategies.

### Optimizing optimal summation

Although we show that the default assumption of the ensemble-free mode of change detection can be problematic, we think that the idea to predict full-set performance based on single-item performance is a prospective tool for strong quantitative measures of visual working memory. However, it needs a further adjustment to correctly incorporate potentially different contributions of each individual item to overall performance. It is not the purpose of this current work to propose a full-fledged modification of this model. However, we would like at least to suggest a direction to elaborate this approach. As Experiment 2 showed, the detectability of a change in an individual item (*d′_i_* in Equations 2a and 2b) is set not only by the difference between the pre-change and the post-change features but also by the direction of change relative to the feature distribution. Hence, a proper prediction for optimal summation or the minimum rule (full-set *d′*_total_ in Equations 2a, 2b, 8, and 9) should take into account *d′_i_-*s measured separately for each item.

There is an important implication of this approach for the conclusion about item dependence on the ensemble or other items. If change detection is indeed performed for each item independently, then all individual *d′*-s are equal, and the predictions for the *d′*_total_ are exactly as in Equation 3. However, if the individual *d′-*s are different, then the optimal *d′*_total_ should differ from that.

## Conclusions

Harrison et al. (2021) suggested a promising approach to the analysis of change-detection performance based on multidimensional SDT. This approach can provide useful insights into how information about individual memoranda is integrated in visual working memory. Having said that, our study points out a set of important caveats about this approach and suggests its further development. Specifically, we showed that direct mapping of one-item sensitivity (*d′*_one_) onto the multidimensional detection space is not sufficient to make straightforward predictions about ensemble-free integration of the set into working memory. Furthermore, we presented some theoretical arguments for why the optimal summation evidence from individual items inherently involves ensemble-based decisions. Overall, when the statistical structure of the memory set and a decision rule are taken into account, the data seem to support the idea that ensemble information is used in visual change detection.

## Appendix

Our simulation aimed to demonstrate how *d′*_total_ should change as a function of the predicting value of *d′*_one_ if the observer does or does not encode ensemble summary statistics in addition to optimal summation of evidence from individual summation. To this end, we simulated the ideal observer's performance in the full-set change detection task with set size 4 where individual item changes, when present, also caused change in either mean or variance of the whole set, as in the experiments by Harrison et al. (2021). Within the same set of trials, we implemented five versions of multidimensional SDT (all of which are individual cases of Equation 5 in the main text): (1) Model 1: only optimal summation (no ensemble memory), (2) Model 2: optimal summation + ensemble statistics with double sampling (different sets of samples are used to encode individual items and ensemble summaries), (3) Model 3: optimal summation + ensemble statistics with single sampling (both individual items and ensemble summary statistics are encoded from the same set of samples), (4) Model 4: optimal summation + ensemble statistics with single sampling and independent memory noise not related to sampling, and (5) Model 5: optimal summation + ensemble statistics with single sampling, independent memory noise, and late noise related to the computation of ensemble statistics. An R code to run these simulations and visualize its results (Figure 1) is available online at the same address as the other online materials.

We have run 200,000 simulations for each input *d′*_one_ ranging from 0 to 1.5 with a step of 0.1. Instead of using physical spaces to assign stimulus features, we directly worked with the arbitrary signal-detection space where all stimulus differences were defined as the distance between corresponding Gaussian distributions in *z*-units (σ = 1 for each distribution). Each such Gaussian distribution was used to simulate sampling from individual items. We arbitrarily assigned a base set of features values of −0.6, −0.2, 0.2, and 0.6. Like in Harrison et al. (2021), we altered the base set two different ways to accomplish change either in mean, or in variance. For mean-change trials, we added a *d′*_one_ to each of the base values, such that the whole distribution shifted to the right but its variance stayed the same. For variance-change trials, we added a −*d′*_one_ to the two negative values of the base set and a *d′*_one_ to the two positive values, such that the mean stayed the same but variance increased. On each trial of the change-present condition, the pre-change role was randomly assigned to either the base, or the altered set and then the remaining set took the post-change role. On each trial of the change-absent condition, either the base set or the altered set was used as both pre-change and post-change.

### Sampling, summarization, and evidence for change

In each trial, we first randomly picked two sets of samples from all pre-change and post-change item distributions. Each set of samples included four numbers, each drawn from one of the four Gaussian distributions corresponding to each item. In Models 1 through 3, as soon as *d′*_one_ reflects change discriminability between the pre-change and post-change samples, that is, sensitivity to the differences between the two samples, the standard deviations of the distributions from which samples were drawn, σ_pre_ and σ_post_, were 1/√2, such that σ^2^_post – pre_ = σ^2^_pre_ + σ^2^_post_ = 1. In Models 4 and 5 with additional memory noise independent from that coming from sampling, we first set an arbitrary value of the memory noise, σ_memory_ such that 0 < σ_memory_ < 1. We assumed that the overall change-detection noise (σ = 1) is a linear combination of sampling noises in both displays (σ_pre_ and σ_pre_) and independent memory noise, such that σ^2^_pre_ + σ^2^_post_ + σ^2^_memory_ = 1. Therefore the sampling noise corrupting each individual pre-change and post change color in Models 4 and 5 was σ^2^_sampling_ = √[(1 – σ^2^_mem_)/2] (assuming σ_pre_ = σ_post_). The first set of samples was used to encode individual feature values in all evidence integration models and for ensemble encoding in the models with ensemble memory and single sampling (Models 3–5). The second set of samples was used for ensemble encoding only in the model with ensemble memory and double sampling (Model 2). We then calculated the average and standard deviation for the pre-change and the post-change sample values (based on the first or the second set of samples).

Evidence for change was calculated both for individual items and for summary statistics. In Models 1 through 3 where all noise in individual representations came from sampling, we subtracted each pre-change sample value from the corresponding post-change value. In Models 4–5, a random number from the memory noise distribution (standard deviation = σ_memory_) was added to the difference between the pre-change and post-change samples. In all models including ensemble summary statistics (Models 2–5), we subtracted the pre-change summaries from post-change summaries, as we did for individual samples. In Model 5, the difference between the summaries was additionally corrupted by the ensemble noise (σ^2^_ensemble_) which accounted for all independent sources of error in ensemble memory independent from sampling.

Because signal-detection analysis requires that the strength of evidence is a unipolar scale (the bigger the value, the stronger the evidence for change presence), we had to deal with the fact that some of the stimuli changed in the negative directions in our arrangement (e.g., some of the individual items changed by −*d′*_one_ ; the same was true for the summary statistics). To transform this bipolar arrangement to unipolar, we flipped the sign of the post-change—pre-change difference every time when the “ground truth” direction of change was negative, both for individual items and for summary statistics

### Evidence integration, decision making, and *d′*_total_

We implemented three evidence integration models. In Model 1, the sum of evidence coming only from individual samples is calculated and used to decide on the response to be made. In Models 2–5 (optimal summation and memory for ensemble statistics), evidence about mean change and standard deviation change were added to the sum of evidence coming from individual items. All five models then used the same algorithm for decision making. We arbitrarily set a decision criterion at *d′*_one_. The model answered “yes” if the cumulative evidence was larger than this criterion, and the model answered “no” otherwise. Proportions of “yes” answers across all change-present (hits) and change-absent (false alarms) trials were then used to calculate the *d′*_total_ (see Equation 1 in the main text) predicted by each model.
